# Greenhouse Gas Inventory of a Typical High-End Industrial Park in China

**DOI:** 10.1155/2013/717054

**Published:** 2013-02-03

**Authors:** Bin Chen, Guoxuan He, Jing Qi, Meirong Su, Shiyi Zhou, Meiming Jiang

**Affiliations:** ^1^State Key Joint Laboratory of Environmental Simulation and Pollution Control, School of Environment, Beijing Normal University, Beijing 100875, China; ^2^Beijing Development Area Co., Ltd., Beijing 100176, China

## Abstract

Global climate change caused by greenhouse gas (GHG) emissions, which severely limits the development of human society and threatens the survival of humanity, has drawn the international community's long-term attention. Gathering the most important production factors in the region, an industrial park usually represents the development level of specific industries in the region. Therefore, the industrial park should be regarded as the base unit for developing a low-carbon economy and reducing GHG emissions. Focusing on a typical high-end industrial park in Beijing, we analyze the carbon sources within the system boundary and probe into the emission structure in view of life-cycle analysis. A GHG inventory is thereby set up to calculate all GHG emissions from the concerned park. Based on the results, suggestions are presented to guide the low-carbon development of the high-end industrial park.

## 1. Introduction

 Global climate change caused by greenhouse gas (GHG) emissions, which severely limits the development of human society and threatens the survival of humanity, has drawn the international society's attention. China has been considered as the largest GHG emitter in the world, and the Chinese government has been taking various measures to reduce GHG emission and mitigate climate change [1–6]. Among them, China is committed to cut the CO_2_ emission per unit of GDP by 40–45% by 2020 against the 2005 level [7]. Within this context, low-carbon economy provides an efficient development path that maximizes the value in a low consumption, low pollution, and low GHG emission mode [8, 9].

Industrial parks are characterized as a clustering of industries designed to meet compatible demands of different organizations within one location [10]. Industrial agglomeration has proven to be vital to the economic growth of developed countries, as well as less-developed ones like China [11, 12]. According to the data from the Ministry of Land and Resources in China 2011, there were 208 nationally designated industrial parks and more than 3000 provincial ones in China. For the industrial land, the average fixed investment was 48.1 million RMB per hectare, and the output was 125.6 million RMB per hectare [13–15]. Taking Beijing as an example, all the industrial parks' contribution to the urban economy accounted for 24%. Collecting most important factors of production in the region, an industrial park usually represents the development level of specific industries in the region. Therefore, the industrial park should be regarded as the base unit for developing industrial economy and also a breakthrough for regional resource allocation and environmental management. With the emergence of low-carbon economy, more and more industrial parks are seeking a low-carbon mode incorporating production, consumption, and resource allocation issues. 

In the industrial park, enterprises of identical or similar industries are not simply spatially concentrated. The administrator must plan the layout, build the public infrastructure, and handle the daily maintenance. Therefore, GHG emissions from industrial parks could be divided into two parts, and the responsibility for emission cutting could be shared by different parties including the enterprises and the administrator of the park. Currently, there have been increasing research interests focused on the GHG emissions from enterprises and industrial sectors, shaping the GHG inventories in expanding scopes, from the combustion of fossil fuel to the whole production chain [16–21]. This means, in spite of the direct emission from energy consumption and chemical process, the inventories also include indirect emission owing to the total supply chain extend to the production gate [22]. In fact, it is necessary to calculate the GHG emission from the perspective of administrators and put forward useful suggestions on carbon reduction, which may supplement the existing literatures and help industrial parks achieve the low-carbon mode.

High-end industrial park is a type of industrial park that usually agglomerates the head offices or research and development centers of high-tech industries. There are few manufacturing productive processes in such industrial parks, so less fossil fuel and fewer raw materials are consumed by the enterprises. As a result, the administrator would take more responsibilities compared to those of the other types of industrial parks. In this paper, a GHG inventory is set up to analyze the life-cycle GHG emissions of a high-end industrial park in Beijing. Based on the results, suggestions are given to guide the low-carbon development of the high-end industrial park.

## 2. Methodology

The life cycle of a high-end industrial park is divided into three stages, that is, construction stage, operation stage, and demolition stage. In the construction stage, the concerned buildings and affiliated municipal facilities are constructed, as well as the garden landscape. The operation stage usually lasts for 40 to 50 years. During this stage, the industrial parks need to consume a large amount of electricity, heat power, and water. Meanwhile, wastewater and solid waste are discharged daily from the buildings and public space. As a general rule, a high-end industrial park has a 50-year term for the land use rights, and then it goes into the demolition stage. In this study, the GHG emission in the demolition stage is estimated based on the size and construction structure of the industrial park.

We consider three types of GHGs, that is, carbon dioxide (CO_2_), methane (CH_4_), and nitrogen oxide (N_2_O). The inventory includes five sorts of GHG emissions sources: (1) direct and indirect emission from the consumption of primary energy and second energy; (2) direct emission from industrial production process; (3)-(4) emission from the production and transportation processes of all the materials and equipment used in the high-end industrial park; (5) emission from the sewage treatment and solid waste disposal processes.

### 2.1. Energy Consumption

Energy consumption is an important source of GHG emissions in the industrial park. As defined in this study, energy consumption processes involved mainly includes fossil fuel combustion, electricity and heat energy production, and transportation vehicles. The estimation of the GHG emission from above processes refers to the 2006 IPCC Guidelines for National Greenhouse Gas Inventories [22].

For the fossil fuel combustion, (1) is given as

(1) $$\begin{array}{c} E_{\text{GHG},i}^{c}=Q_{i}^{c}\times {\text{EF}}_{\text{GHG},i}^{c}, \end{array}$$

where  *E*_(GHG,*i*)_^*c*^ is the emissions of a given GHG by type of fuel (kg), *Q*_*i*_^*c*^ is the amount of fuel combusted (TJ), and  EF_GHG,*i*_^*c*^ is the emission factor of a given GHG by type of fuel (kg GHG/TJ).

For the electricity and heat energy productionprocesses,if the power comes from the sectors outside the industrial park, the concerned GHG emission can be calculated according to the average energy consumption level of the local sector. If the power is produced by the enterprise just in the industrial park, the concerned GHG emission can be estimated as zero to avoid the repeated calculation based on the fossil fuelofcombustion process. Equation (2) is presented to calculate the GHG emission from electricity and heat energy productionprocesses as

(2) $$\begin{array}{c} E_{\text{GHG}}^{e}=Q^{e}\times {\text{EF}}_{\text{GHG}}^{e}, \end{array}$$

where *E*_GHG_^*e*^ is the emissions of a given GHG by electricity or heat (kg),  *Q* ^*e*^ is the amount of electricity or heat produced (kWh or TJ), and EF_GHG_^*e*^ is the emission factor of a given GHG by electricity or heat (kg GHG/kwh or kg GHG/TJ).

As for the transportation process, it refers to the transportation of the energy resources, raw materials, instruments and equipment from the origin to the industrial park. The GHG emissions from the transportation process can be estimated from the fuel consumed:

(3) $$\begin{array}{c} E_{\text{C}{\text{O}}_{2}}^{t}=\sum_{i}\left(Q_{i}^{t}\times {\text{EF}}_{\text{C}{\text{O}}_{2},i}^{t}\right), \end{array}$$

where  *E*_CO_2__^*t*^ is the emissions of CO_2_ (kg),  Q_*i*_^*t*^ the fuel consumed (TJ), and  EF_CO_2_,*i*_^*t*^ is the emission factors (kg CO_2_/TJ), or calculated by the distance covered by the vehicles as

(4) $$\begin{array}{c} E_{\text{N}{\text{H}}_{4},{\text{N}}_{2}\text{O}}^{t}=\sum_{a,b,c,d}\left(L_{a,b,c,d}\times {\text{EF}}_{a,b,c,d}^{t}\right)+\sum_{a,b,c,d}C_{a,b,c,d}, \end{array}$$

where *E*_NH_4_,N_2_O_^*t*^ is the emissions of CH_4_ or N_2_O (kg), *L*_*a*,*b*,*c*,*d*_ is the distance travelled during thermally stabilized engine operation phase for a given mobile source activity (km), EF_*a*,*b*,*c*,*d*_^*t*^ is the emission factor (kg/km), *C*_*a*,*b*,*c*,*d*_ is the emissions during warm-up phase (cold start)(kg), *a* is the fuel type (e.g., diesel, gasoline, natural gas, and LPG), *b* is the vehicle type, *c* is the emission control technology (such as uncontrolled, catalytic converter, etc.), and *d* is the operating conditions (e.g., urban or rural road type, climate, or other environmental factors).

In general, the first approach (fuel sold) is appropriate for CO_2_ while the second (distance travelled by vehicle type and road type) is suitable for CH_4_ and N_2_O.

### 2.2. Industrial Production

The GHG emissions from industrial production processes of specific products are mainly due to the physical or chemical reaction rather than the combustion of fossil fuels. In an industrial park, the GHG type and amount depend on the leading industry type, and the accounting method varies accordingly. According to the activity data obtained and the applicable scope of emission factors, there are two scales to calculate the emission from the industrial production process: an industry or a product. If it is an industry, the calculation is less precise. The activity data can be the economic scale (such as the GDP) or the production scale (such as the production output), and the emission factors can be the recommended ones by IPCC or the average level of the specific countries or territories. Regarding a product, the calculation must focus on the supply chain of the product, even narrowed down to production device and working conditions. For a high-end industrial park in this study, there is almost no real supply chain of product.

### 2.3. Material Input

Similar to the electricity and heat energy production processes, repeated calculations should also be avoided when studying the GHG emission due to the material input. There is a development trend of the industrial parks that more and more industrial parks will generate internal logistics networks, and then the products, by-products, or waste of one enterprise will soon be used by another enterprise as raw material or energy. Therefore, in this study, the objects are the materials coming from the outside, and the calculation will cover all the GHG emission during the production and transportation processes that is usually defined as “from cradle to gate.” GHG emission of this part could be calculated by

(5) $$\begin{array}{c} E_{\text{GHG},i}^{m}=Q_{i}^{m}\times {\text{EF}}_{\text{GHG},i}^{m}, \end{array}$$

where *E*_GHG,*i*_^*m*^ is the emissions of GHG during the production and transportation processes of type of material (kg), *Q*_*i*_^*m*^ is the amount of material input, and EF_GHG,*i*_^*m*^ is the emission factor.

### 2.4. Equipment Employment

Some equipment will be applied directly by enterprises for industrial production activities, such as blast furnace and converter that are essential to iron and steel industry; and the other equipment will be used to maintain the daily operation of the industrial parks such as water pump, draft apparatus, and so forth. Similar to the material input process, the calculation of equipment employment will also cover the “from cradle to gate” GHG emission. There are two methods to obtain the emission factors of equipment: (1) one is based on the input-output between the equipment production sector and other sectors, and the result will be in the form of GHG emission per unit currency, as described in [23, 24]; (2) the other one is to analyze the raw materials dosage and processing energy consumption of a specific equipment, and the result will be in the form of GHG emission per piece of equipment, as described in references [25–27].

### 2.5. Sewage Treatment and Solid Waste Disposal

The conventional disposal of solid waste is landfill, composting, and burning. The landfill and composting will mainly generate CH_4_, and burning produces CO_2_. Currently, landfill is the most popular method in the Chinese cities to dispose of the solid waste, and the technology of GHG collection under such condition is developing rapidly and being promoted widely. In this study, the GHG emission from solid waste disposal process will be calculated using the first-order attenuation method as recommended by IPCC as

(6) $$\begin{array}{c} E_{\text{C}{\text{H}}_{4}}^{s}=\left(\sum_{x}E_{\text{C}{\text{H}}_{4},x,T}^{s}-R_{T}\right)\times \left(1-{\text{OX}}_{T}\right), \end{array}$$

where *E*_CH_4__^*s*^ is the emission of CH_4_ from solid waste disposal (kg),  *E*_CH_4_,*x*,*T*_^*s*^ is the emission of CH_4_ from a type of solid waste disposal (kg),  *R*_*T*_ is the recovery of CH_4_ (kg), and OX_*T*_ is the oxidation factor (%).

The usual sewage anaerobic treatment will discharge CH_4_ and N_2_O, where the amount depends on the contents of biodegradable organics and nitrogenous substance in the sewage water. The calculation method recommended by IPCC is chosen to estimate the GHG emission from the sewage treatment process as

(7) $$\begin{array}{c} E_{\text{C}{\text{H}}_{4}}^{w}=\text{TOW}\times {\text{EF}}_{\text{C}{\text{H}}_{4}}^{w}-R, \end{array}$$

where *E*_CH_4__^*w*^ is the emission of CH_4_ from sewage treatment (kg), TOW the total organic degradable material in wastewater (kg), EF_CH_4__^*w*^ the emission factor (kg/kg),  *R* is the recovered CH_4_ (kg) and

(8) $$\begin{array}{c} E_{{\text{N}}_{2}\text{O}}^{w}={\text{N}}^{w}\times {\text{EF}}_{{\text{N}}_{2}\text{O}}^{w}\times \frac{44}{28}, \end{array}$$

where *E*_N_2_O_^*w*^ is the emission of N_2_O from sewage treatment (kg), N^*w*^ is the nitrogen in effluent (kg),  EF_N_2_O_^*w*^ is the emission factor (kg/kg), and44/28 is the conversion factor of kg N_2_O-N into kg N_2_O.

## 3. Case Study

### 3.1. Data Sources

The concerned industrial park is located in the southwest of Beijing. It is a typical high-end industrial park known for its good environmental quality and high-end industry concentration. The park has an area of 119235.6 m^2^. The building density is 31.62%, while the landscaping ratio is 41%, which means 1 square meter of park area is covered by 0.3162 square meters of building and 0.41 square meters of landscape. All the data in our case industrial park are obtained by field research, while the emission factors from the public data sources. In order to compare the results, all the GHG emissions in this study are converted to the form of CO_2_-eq based on the global warming potential (GWP) recommended by IPCC [28].

#### 3.1.1. Energy Consumption

The emission factors of primary energy are specific for China, which are referenced to the default emission factors by IPCC and the average lower heat values in China's energy statistics yearbook [29]. The emission factors of the secondary energy (such as the electricity and heat) are obtained based on the amount of primary energy consumed by the electricity and heat production sector in Beijing. The basic data can be found in the reports of the National Development and Reform Commission and* Beijing Statistical Yearbook* [30].

#### 3.1.2. Industrial Production

The industrial park is designed with high-end positioning that is a well-operated cluster of the head offices or research and development centers of high-tech industries. Thus, the GHG emission of industrial production process in our case is neglected.

#### 3.1.3. Material Input

As discussed in Section  3.1.2, there are few industrial production processes in such high-end industrial parks. We thus have the hypothesis that the raw material input necessary for industrial production can be ignored. As a result, this study focuses on the GHG emission from the construction materials input during the construction stage. The emission factors of the construction materials can be divided into 3 types [31–43].

*Type* 1 The products are easy to decide their boundaries and technological processes to choose the precise matching emission factors.

*Type* 2 The products are not well studied by life-cycle analysis but the production technological processes are clear. We can obtain the emission factors based on the amount of energy consumption and raw material input, on the condition that these energy consumption and raw material input are of Type 1.

*Type* 3 The products have never been studied. We have to select an alternative that has similar production process, raw material, and function, with an assumption that the emission factors of them are the same.

#### 3.1.4. Equipment Employment

The method with which we obtained the emission factors of the equipment is the same with the material input. However, the equipment has a more complicated production process and various specifications and types, so it is difficult to make clear the exact emission factors of each piece of equipment [44–47]. As a result, the majority of equipment belongs to Type 3, and the uncertainty of the calculation results is increased.

#### 3.1.5. Sewage Treatment and Solid Waste Disposal

According to the investigation, all the sewage generated by the case industrial park is discharged into the municipal drainage sewage pipe network, so we assume that the emission factors of the sewage from the industrial park are the same as the average of the municipal sewage in Beijing. With reference to the literatures [48, 49], the emission factors of the sewage treatment process are (5.61*E* − 03) kg CO_2_/kg, (8.89*E* − 08) kg N_2_O/kg, and (1.59*E* − 02) kg CH_4_/kg. 

Similarly, the emission factors of the solid waste disposal process are assumed to be the same as the average level in Beijing. With reference to the literatures [50], the CH_4_ correction factor (MCF) is 1.0, the dissolved organic carbon (DOC) 6.5%, the fraction of DOC dissolved (DOC_f_) 0.5, the CH_4_ volume fraction of the landfill gas 0.5, and the oxidation factor (OX_T_) 0.1.

### 3.2. Results and Discussion

The GHG emission of each stage in the life cycle of the industrial park is calculated based on the proposed method. The overall GHG emission of the life-cycle is 1872177 t CO_2_-eq. The construction stage takes up 4.546%, which amounts to 85105.82 t CO_2_-eq GHG emission with an intensity of 801.69 kg CO_2_-eq/m^2^; the demolition stage takes up 0.102%, contributing 1917.3 t CO_2_-eq GHG emission with an intensity of 18.06 kg CO_2_-eq/m^2^. As can be seen, the operation stage contributes the majority of GHG emission, which achieves a proportion of 95.352%. The GHG emission amount of operation stage is 37717.18 t CO_2_-eq, with the intensity being 355.29 kg CO_2_-eq/m^2^. 

**Figure 1:**
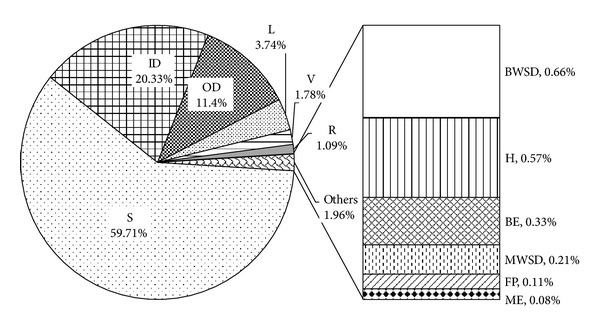
GHG emission from construction material of construction stage.

The construction stage of the industrial park is decomposed into 12 steps with different functions, which are structure (S), indoor decoration (ID), outdoor decoration (OD), building electric (BE), building water supply and drainage (BWSD), heating (H), ventilation (V), fire protection (FP), road (R), municipal electric (ME), municipal water supply and drainage (MWSD), and landscaping (L). During the construction stage, GHG emission from construction material input process is accounted to be 82509 t CO_2_-eq, which takes the 96.95% part of the stage. The contribution of each step to the amount can be found in Figure 1. As it is shown, the top three emission sources are S (59.71%), ID (20.33%), and OD (11.40%) and then L (3.74%), V (1.78%), and R (1.09%). The other six steps only take up the proportion of less than 1%.

**Figure 2:**
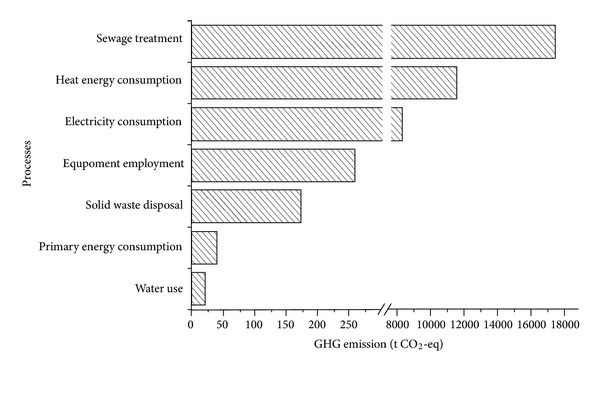
GHG emission of operation stage.

The operation stage should be of the focus of GHG emission reduction in the industrial park for its significant contribution. In order to get more specific and meaningful results, the overall GHG emission of this stage is further decomposed into seven processes. As shown in Figure 2, the processes of sewage treatment, heat energy consumption and electricity consumption should be paid more attention, which contributes to 98.69% of the operation stage emission. 

**Figure 3:**
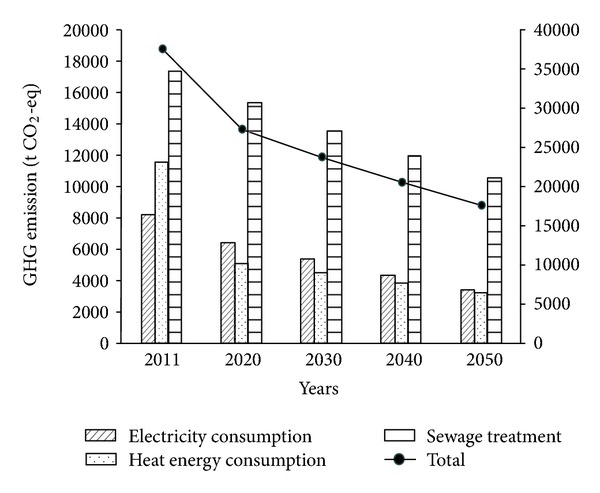
GHG emission of operation scenarios of different strategies.

For the operation stage, some strategies are adopted to change the energy consumption and sewage treatment as shown in Table 1. The GHG emission of operation stage will decline as shown in Figure 3. The overall emission of operation stage will be reduced to 27443.58 t in 2020 and 17711.66 t in 2050, which are just 72.76% and 46.96% of those in 2011.

## 4. Conclusions

For a high-end industrial park, the GHG emission of the construction stage is very intensive with significant environmental impacts, which is a key point to be reckoned with by the administrators. Based on the calculation results, we can control the GHG emission of construction stage in the following ways.

- *Using local construction materials to reduce the GHG emission from the transportation processes of the construction materials*. Most construction materials are of a large size and usually in high demand. Thus, there will be a considerable quantity variance of GHG emission between different transport distances. Employing the local construction materials can not only save the transportation cost and transportation time, but also reduce the GHG emissions, which may achieve much more benefits in economy and environment.
- *Employing the low-carbon and regeneration construction materials instead of the traditional ones to reduce the GHG emission from the upstream production process and downstream disposal*. After the quantitative evaluation of performance and cost, decision makers may prefer the low-carbon and regeneration construction materials. The low carbon and regeneration characteristics imply that these materials consume less energy and fewer resources during the production processes compared to the traditional materials, thereby having a better performance and lower price.
- *optimizing the construction progress to promote a safe and low-carbon form of construction engineering*. The arrangement of the construction schedule can be optimized to ensure the project to be finished on time. For example, less night work can avoid unnecessary power consumption and noise pollution.

The administrators of industrial parks should take the responsibilities to reduce the GHG emission during the operation stage, which normally lasts for about 40 to 50 years. As implied by the accounting result, more attention should be paid to the processes of sewage treatment, heat energy consumption, and electricity consumption when controlling the GHG emission of this stage. For example, the industrial park in our case produces 40,000 t sewages every year, and the treatment process of the sewage will result in a GHG emission of 17373.46 t CO_2_-eq. Therefore, the administrator can control the emission by bringing in water saving technology and building the recovery system of waste water. 

## Figures and Tables

**Table 1:** Scenario analysis of operation stages.

Emission source	Parameter	2010	2020	2030	2040	2050
Electricity consumption	Emission factor (g CO_2_-eq/kWh)	996.36	862.52	759.91	654.78	552.17
Heat energy consumption	Emission factor (kg CO_2_-eq/GJ)	101.43	44.88	39.27	33.66	28.05
Sewage treatment	Emission factor (g CO_2_-eq/kg sewage)	435.45	413.20	392.21	372.22	353.21
